# Characterization of *FGFR1* Locus in sqNSCLC Reveals a Broad and Heterogeneous Amplicon

**DOI:** 10.1371/journal.pone.0149628

**Published:** 2016-02-23

**Authors:** Claire Rooney, Catherine Geh, Victoria Williams, Johannes M. Heuckmann, Roopika Menon, Petra Schneider, Katherine Al-Kadhimi, Michael Dymond, Neil R. Smith, Dawn Baker, Tim French, Paul D. Smith, Elizabeth A. Harrington, J. Carl Barrett, Elaine Kilgour

**Affiliations:** 1 AstraZeneca, Oncology Innovative Medicines, Alderley Park, Macclesfield, United Kingdom; 2 AstraZeneca, Personalised Healthcare and Biomarkers Innovative Medicines, Alderley Park, Macclesfield and Melbourn, Cambridge, United Kingdom; 3 Blackfield AG, Cologne, Germany; 4 AstraZeneca, Discovery Sciences Innovative Medicines, Alderley Park, Macclesfield, United Kingdom; 5 AstraZeneca Oncology Innovative Medicines, Gatehouse Park, Waltham, Massachusetts, United States of America; Advanced Centre for Treatment, Research and Education in Cancer, Tata Memorial Center, INDIA

## Abstract

*FGFR1* amplification occurs in ~20% of sqNSCLC and trials with FGFR inhibitors have selected *FGFR1* amplified patients by FISH. Lung cancer cell lines were profiled for sensitivity to AZD4547, a potent, selective inhibitor of FGFRs 1–3. Sensitivity to FGFR inhibition was associated with but not wholly predicted by increased *FGFR1* gene copy number. Additional biomarker assays evaluating expression of FGFRs and correlation between amplification and expression in clinical tissues are therefore warranted. We validated nanoString for mRNA expression analysis of 194 genes, including FGFRs, from clinical tumour tissue. In a panel of sqNSCLC tumours 14.4% (13/90) were *FGFR1* amplified by FISH. Although mean *FGFR1* expression was significantly higher in amplified samples, there was significant overlap in the range of expression levels between the amplified and non-amplified cohorts with several non-amplified samples expressing FGFR1 to levels equivalent to amplified samples. Statistical analysis revealed increased expression of *FGFR1* neighboring genes on the 8p12 amplicon (*BAG4*, *LSM1* and *WHSC1L1*) in FGFR1 amplified tumours, suggesting a broad rather than focal amplicon and raises the potential for codependencies. High resolution aCGH analysis of pre-clinical and clinical samples supported the presence of a broad and heterogeneous amplicon around the FGFR1 locus. In conclusion, the range of *FGFR1* expression levels in both *FGFR1* amplified and non-amplified NSCLC tissues, together with the breadth and intra-patient heterogeneity of the 8p amplicon highlights the need for gene expression analysis of clinical samples to inform the understanding of determinants of response to FGFR inhibitors. In this respect the nanoString platform provides an attractive option for RNA analysis of FFPE clinical samples.

## Introduction

Lung cancer represents the leading cause of cancer-related deaths [1] and remains one of the most challenging diseases to treat. Non-small cell lung cancer (NSCLC) is subdivided into histological subtypes, adenocarcinoma, large cell carcinoma and squamous cell carcinoma and together these represent about 85% of lung cancer cases. Genomic characterization of NSCLC has identified actionable alterations that have lead to the adoption of targeted therapies as standard of care in defined patient populations. EGFR inhibitors are approved for EGFR mutation positive tumours and anaplastic lymphoma kinase inhibitors are approved for EML4-ALK fusion positive tumours [2–4]. However, these genetic events are limited to the adenocarcinoma subtype and until the recent approval of the immunotherapy nivolumab for PD-L1 positive cancers no targeted therapies were approved in the squamous subtype to date [5]. In recent years, several therapeutic targets were identified as altered in squamous NSCLC (sqNSCLC) through mutation or amplification including *FGFR1* amplifications which that been identified in ~20% of sqNSCLC cases [6, 7].

The FGF/FGFR signalling axis is comprised of 18 ligands, which exert their actions via 4 highly conserved trans-membrane tyrosine kinase receptors. FGF/FGFR signalling plays an important role in normal organ, vascular and skeletal development, and in the homeostatic control of phosphate and bile acids [8]. FGFR signalling is deregulated in many tumour types through amplification, fusion or mutation of the receptor [9]. In pre-clinical models of sqNSCLC *FGFR1* amplification confers sensitivity to AZD4547, a potent and selective inhibitor of FGFRs 1–3 [10]. This observation and others led to the initiation of several trials of FGFR targeting agents in sqNSCLC including NVP-BGJ398 (NCT01004224) and AZD4547 (NCT00979134).

As data emerges from these trials it is clear that although some patients are deriving clinical benefit from treatment, the rate of clinical responses is lower than predicted by the pre-clinical studies [11, 12]. *FGFR1* is located on chromosome 8p and characterization of the 8p amplicon in sqNSCLC has revealed that the *FGFR1* gene lies at the centre of the amplicon in only 25–30% of cases [13], raising the potential for co-amplification and expression of neighbouring genes. Herein we have undertaken multiple biomarker analyses of pre-clinical cell lines and sqNSCLC tissues to develop our understanding of molecular predictors and identify options to further refine the patient selection strategies.

## Materials and Methods

### Cell lines and tissue samples

NCI-H226, NCI-H2286, NCI-H520, NCI-H596, SKMES-1, SW900, NCI-H2170, DMS114, NCI-H1703, NCI-H1869 and Calu-3 cells were from ATCC. HCC-15 cells were from DSMZ. LUDLU-1 cells were from ECCAC. RERF-LC-SQ1, LK-2 and EBC-1 cells were from JCRB. All cells were cultured in RPMI supplemented with 10% foetal bovine serum and 1% L-glutamine. Cells were maintained in 5% CO_2_ at 37°C. Ninety NSCLC tissues were purchased from Asterand. Prior to processing, each sample was reviewed by an internal certified pathologist to confirm disease diagnosis and verify tumour content.

### Cell proliferation and clonogenic assays

*In vitro* GI_50s_ for the cell panel were calculated as the concentration of AZD4547 required to control cell growth by 50% in a 72-h period, as determined by the colorimetric 3-(4,5-dimethylthiazol-2-yl)-5-(3-carboxymethoxyphenyl)-2-(4-sulfophenyl)-2*H*-tetrazolium assay.

For clonogenic assays, cells were syringed and counted and adjusted to between 400 and 2000 cells per well of a 6 well plate. 2.5mls of each cell line was seeded into 2 x 6 well plates and left to adhere over night. AZD4547 was dosed at 1μM, 0.3μM, 0.1μM, 0.03μM, 0.01μM, DMSO directly onto the cells. The cells were re-dosed every 7 days for 21 days. The colonies were fixed in ice cold 80% ethanol and stained with 1% crystal violet in 15% methanol and counted on Gelcount colony counter.

### RNA extraction

Cell lines were extracted using the RNeasy kit (QIAGEN). FFPE tissues were extracted using the RNeasy FFPE extraction kit (QIAGEN). RNA quantity and quality were assessed by Nanodrop 2000 and RNA 6000 Nano kit (Agilent, Santa Clara, CA), respectively. Protocols were followed according to manufacturer’s instructions.

### *FGFR1* fluorescent in situ hybridisation

FISH probes were provided by Dako (non-commercial kit) as a mix of Texas Red-labelled *FGFR1* DNA probes and fluorescein-labelled *CEN-8* PNA probes. FISH was conducted on 4-μm dewaxed and dehydrated formaldehyde-fixed, paraffin-embedded (FFPE) sections. Sections were incubated in Dako pre-treatment solution at 95°C for 10 minutes. Following pre-treatment, slides were incubated with pepsin solution at 37°C for 3 minutes. Sections and probes were co-denatured at 82°C for 5 minutes and then hybridised at 45°C for 14–20 hours. Following a post-hybridisation wash in DAKO stringency wash buffer, sections were mounted with Dako fluorescence mounting medium. *FGFR1* gene and *CEN-8* signals were observed using a fluorescence microscope equipped with the appropriate filters. Enumeration of the *FGFR1* gene and chromosome 8 was conducted by microscopic examination of 50 tumour nuclei, which yielded a ratio of *FGFR1* to CEN-8. Tumours with *FGFR1* to CEN-8 ratio ≥2 or presence of ≥10% gene cluster were defined as amplified.

### Array comparative genomic hybridization

aCGH was performed on cell lines using Agilent 244K arrays (G4411B) following the manufacturer’s protocol for enzymatic labelling. In brief 1.5–3μg of genomic DNA was labelled using the Agilent Genomic DNA Labelling Kit (5188–5309). The samples were labelled with Cy5 and co-hybridized with a pool of male reference DNA labelled with Cy3 (Promega G1471). Following a post-hybridization wash, the arrays were scanned on an Agilent scanner and analyzed with Feature extraction 9.1 software. The aCGH data was analyzed using CGH Analytics 3.4 software (Agilent).

### DNA extraction

Genomic DNA was extracted from 2 x 5μM FFPE sections/sample using Qiagen AllPrep (Qiagen, Cat nr 80234) according to the manufacturer's instructions. Macrodissection ensured DNA was only extracted from tumour regions. DNA was quantified against a standard curve of human genomic DNA (Roche, Cat nr 11691112001) using commercially available, predesigned TaqMan *RNaseP* Assay (Life Technologies, Cat nr 4316838). qPCR reactions were performed in a total volume of 10 μl consisting of 5 μl of Universal Mastermix (2×) (Life Technologies, Cat nr 4324018), 0.5 μl of RnaseP assay, 2 μl of DNA and 2.5 ul nuclease free water. Following the manufacturer's instructions, all qPCR reactions were run in triplicate on an ABI 7900HT instrument (Applied Biosystems) and thermal cycling conditions were 95°C, 10 min followed by 40 cycles of 95°C for 15 s and 60°C for 1 min.

### qPCR copy number assays

Quantitative PCR analysis of *FGFR1* gene content was performed using commercially available, predesigned TaqMan Copy Number Assays (Assay IDs: Hs02882334_cn, Hs06184858_cn and Hs00770365_cn, each consisting of a pair of unlabelled primers and a FAM labelled, MGB probe) and the *RNase P* Copy Number Reference Assay, with a VIC-labelled TAMRA probe (all from Life Technologies). qPCR reactions were performed in a total volume of 10 μl containing 5 μl Taqman Genotyping Master Mix (2x) (Life Technologies, Cat nr 4371355), 0.5 μl of CNV assay, 2ul DNA and 2.5 μl nuclease free water. Following the manufacturer's instructions, all qPCR reactions were run in quadruplicate on an ABI 7900HT instrument (Applied Biosystems) and thermal cycling conditions were 95°C, 10 min followed by 40 cycles of 95°C for 15 s and 60°C for 1 min. DNA copy number levels were measured relative to the *RNase P* reference gene and normalized to human genomic DNA (Roche, Cat nr 11691112001).

### Western blot

Western blotting was performed using standard SDS–PAGE procedures. In brief, cells were lysed with RIPA buffer on ice. Total proteins were separated on a 4–12% Bis–Tris gel, Invitrogen (Paisley, UK) and transferred to immunoblotting membranes. Membranes were blocked in 5% (w/v) non-fat milk phosphate buffered saline+Tween 20 (3.2 mM Na_2_HPO_4_, 0.5 mM KH_2_PO_4_, 1.3 mM KCl, 135 mM NaCl, 0.05% Tween 20, pH 7.4) and then probed with primary antibodies overnight at 4°C. After washing and incubation with secondary antibodies, detected proteins were visualized using the horseradish peroxidase Western Lightning substrate according to the manufacturer’s instructions (Perkin Elmer, Buckinghamshire, UK). Antibodies used for western blot were anti-FGFR1 (Epitomics) and GAPDH (Sigma).

### nanoString analysis

nCounter data were normalized through an internally developed Pipeline Pilot Tool (NAPPA, publicly available on the Comprehensive R Archive Network, CRAN, Harbron & Wappett (2014) R package: NAPPA http://CRAN.R-project.org/package=NAPPA). In brief, data were log_2_ transformed after being normalized in two steps: raw nanoString counts were first background adjusted with a Truncated Poisson correction using internal negative controls followed by a technical normalization using internal positive controls. Data was then corrected for input amount variation through a Sigmoid shrunken slope normalization step using the mean expression of housekeeping genes. A transcript was designated as not detected if the raw count was below the average of the 8 internal negative control raw counts plus 2 standard deviations reflecting approximately a 95% confidence interval.

### Statistical analysis

For each gene, the amplified and non-amplified cohort means were compared using two-sided t-tests, assuming equal variability. A Storey false discovery rate correction was applied to the p-values from the t-tests to give corresponding *q*-values [14]. The *q*-values were used to identify statistically significant changes between the amplified and non-amplified cohorts. The statistical analysis was carried out using the SAS and R software.

Spearman correlation coefficients and corresponding *p-*values were used to evaluate association between copy number and gene expression. Genes which were below the limit of detection (LOD) for >50% of samples were excluded from the spearman correlation analysis.

### Custom array CGH

Probes for aCGH were designed based on copy number data generated from primary squamous cell lung cancer samples and arrays were ordered from Agilent (4x180k arrays). The overall number of data probes was 132,516, plus normalization-, replication- and control-probes covering the 180k probe array. Raw data, after hybridization and readout, was extracted using the Cytogenomics Software (Agilent). Raw copy numbers were computed by the ratio between the intensities of the Cy3 and Cy5 color- channels of each probe. Next, the baseline was adjusted such that the genome-wide average over the raw copy numbers yields two. Finally, raw copy numbers were segmented by using the circular-binary segmentation algorithm [15].

Histograms were generated using R (version 3.0.1), a free software environment available at http://www.r-project.org.

### RT-PCR

100ng of RNA was converted to cDNA using Superscript vilo cDNA synthesis kit (Invitrogen). PCR reactions were performed in a total volume of 10 μl containing Taqman gene expression master mix (Life Technologies), 0.5 μl of gene expression assay, 1ul cDNA and 3.5 μl nuclease free water. Following the manufacturer's instructions, all PCR reactions were run on an ABI 7900HT instrument (Applied Biosystems). Gene expression assays were purchased from Applied Biosystems: FGFR1 (Hs00915142); FGFR3 (Hs00179829_m1); FGFR2 (Hs01552926_m1); FGF2 (HS00266645_m1). Gene expression was normalized to the average of two housekeeper controls, PGK1 (HS99999905_m1) and ACTB (HS00357333_g1).

### FGFR1 immunohistochemistry

Tissues were sectioned (5μM) onto glass slides, dewaxed and rehydrated. All incubations were performed at room temperature and TBS containing 0.05% Tween (TBST) used for washes. Antigen retrieval was performed in pH 6 retrieval buffer (S1699, Dako) at 110°C for 5min in a RHS-1 microwave vacuum processor (Milestone), then peroxidase activity (3% hydrogen peroxide for 10min), endogenous biotin (Vector, SP-2002) and non-specific binding sites (Dako, X0909) blocked. 1:50 FGFR-1 antibody (Epitomics 2144–1), in antibody diluent (Dako, S0809), was applied to sections for 1hr then the Vectastain Elite ABC kit (Vector, PK-6101) was added as instructed. Sections were washed and developed in diaminobenzidine for 10min (Dako, K3466) then counterstained with Carazzi’s hematoxylin.

## Results

### *FGFR1* amplification and expression predict sensitivity to AZD4547 in a lung cancer cell line panel

**Fig 1:**
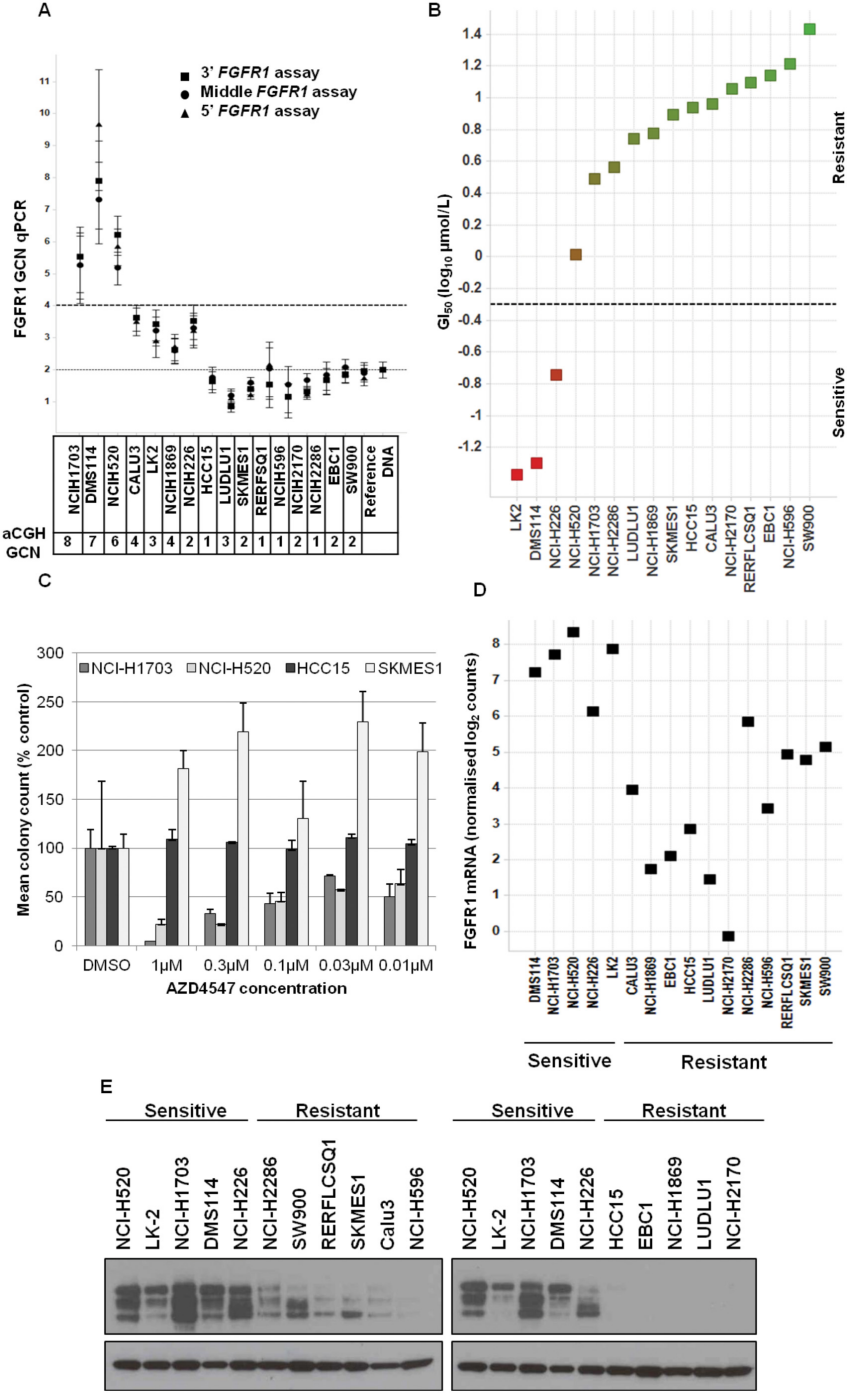
Characterization of *FGFR1* amplification, expression and sensitivity to AZD4547 in lung cancer cell lines. A) *FGFR1* copy number was determined in a lung cell line panel by qPCR. Three different assays targeting 5’, 3’ and middle regions of the gene are shown. Bars: stdev; Table reflects aCGH *FGFR1* copy number for each cell line. B) Sensitivity to FGFR inhibition by AZD4547 treatment was determined by MTS proliferation assay. Sensitive cells lines were defined by GI_50_<200nM. C) Sensitivity to FGFR inhibition by AZD4547 was determined in a long term clonogenic assay in cell lines indicated. Mean colony count was normalised to DMSO control for each cell line. D) Expression of FGFR1 mRNA was determined in AZD4547 sensitive and resistant cell lines by nanoString analysis. E) Expression of FGFR1 protein was determined by western blot in indicated cell lines.

Clinical trials with FGFR inhibitors in sqNSCLC have selected patients with amplifications in *FGFR1*, as determined by FISH. In preclinical explant models of lung cancer, *FGFR1* amplification conferred sensitivity to AZD4547 treatment [10]. We further assessed the relationship between sensitivity to FGFR inhibition and *FGFR1* copy number in a panel of lung cancer cell lines, comprising of 15 sqNSCLC cell lines and the small cell lung cancer cell line DMS114. *FGFR1* copy number was assessed in the cell panel by both qPCR and array comparative genomic hybridisation (aCGH). Three different *FGFR1* qPCR copy number assays were employed targeting the 5’, 3’ and middle regions of the gene. Gene copy number (GCN) was determined relative to RNAseP as a control gene and normalised to reference DNA. DMS114, NCI-H1703 and NCI-H520 cells were found to have an *FGFR1* amplification (defined by inferred GCN >4) both by qPCR and aCGH analysis. Calu-3, NCI-H1869, LK-2 and NCI-H226 cells had low-level increases in *FGFR1* inferred GCN by qPCR, but did not reach the cut off of inferred GCN>4 for amplification. Further, this increase in inferred GCN was not confirmed by aCGH for NCI-H226 cells (Fig 1A). To establish the relationship between *FGFR1* amplification and sensitivity to FGFR inhibition, we profiled this panel of cell lines for sensitivity to AZD4547 in an MTS assay. Of the amplified cell lines, only DMS114 cells were sensitive to AZD4547 treatment. NCI-H1703 and NCI-H520 had IC_50_ values >1μM in the MTS assay (Fig 1B). However, these cells lines were reported as sensitive to FGFR1-targeting shRNA in clonogenic assays [16]. We therefore investigated the sensitivity of these cell lines to AZD4547 treatment in a clonogenic assay. Using this endpoint, NCI-H1703 and NCI-H520 were determined as sensitive to AZD4547, whereas HCC15 and SKMES1 cells, which were also insensitive to AZD4547 in the MTS assay, remained insensitive to AZD4547 treatment in the clonogenic assay (Fig 1C and S1 Fig). The altered dependence of these cells in short term versus long term proliferation assays may reflect an increased dependence in autocrine FGF/FGFR signalling in long term assays. Taken together, these data demonstrate that *FGFR1* amplification predicts for sensitivity to AZD4547 treatment within the cohort of lung cancer cell lines. However, sensitivity to FGFR inhibition is not exclusively predicted for by *FGFR1* amplification status. Notably, two non-amplified cell lines, LK-2 and NCI-H226, were also sensitive to AZD4547 with IC_50_ values <200nM (Fig 1B).

We therefore profiled the cell panel for expression of *FGFR1* to determine whether *FGFR1* expression would discriminate sensitive from insensitive cell lines. *FGFR1* expression profiling by nanoString analysis revealed that all sensitive cell lines had relatively high expression of FGFR1 mRNA (Fig 1D). The mRNA expression data was correlated with protein expression by western blot analysis, demonstrating that the sensitive lines expressed more FGFR1 protein than the insensitive lines (Fig 1E). *FGFR2* or *FGFR3* expression was not correlated with sensitivity in the lung cell line panel (S2 Fig). Taken together, these data suggest that *FGFR1* amplified cell lines have high FGFR1 expression and are sensitive to AZD4547 treatment and when *FGFR1* expression is increased through a mechanism independent of amplification, sensitivity to FGFR inhibition can also occur.

### Validation of nanoString for gene expression analysis on FFPE sqNSCLC tissues

**Fig 2:**
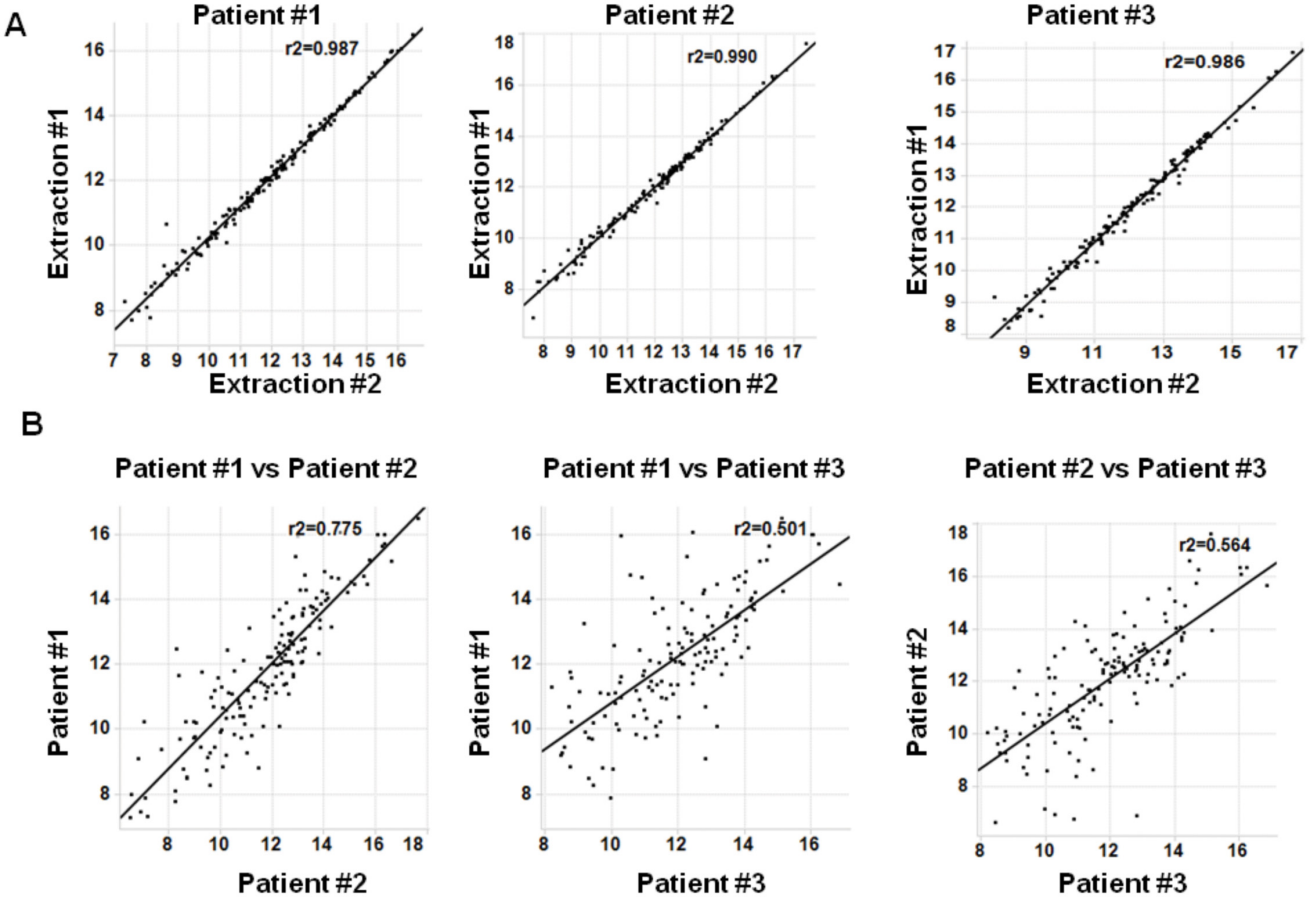
Assessment of inter- and intra-patient variability of nanoString gene expression profiles in sqNSCLC tissues. A) Intra-tumoural variability was assessed using mRNA extracted from different regions of three sqNSCLC tissues. Extraction #1 and extraction #2 were from sections 190μm apart. B) Inter-patient variability between indicated samples was assessed. Input RNA: 400ng. Normalized log_2_ values of 194 genes are shown. Line of identity between samples is shown. r^2^ = Pearson correlation co-efficient.

The preclinical data presented here suggests that in clinical trials where patients are selected on *FGFR1* amplification, additional biomarkers to assess FGFR1 expression are warranted. We therefore evaluated the nanoString platform for gene expression profiling in FFPE sqNSCLC tissues. mRNA was extracted from a 5μm section of sqNSCLC tumour tissue. As shown previously, good correlation was observed between Taqman RT-PCR and nanoString [17–19] (S3 Fig). To determine the reproducibility of nanoString gene expression profiles within a tumour sample RNA was isolated from sections that were 190μm apart within the FFPE tumour from three different patients. For each patient tested intra-tumoural variability in the expression of 194 genes by nanoString analysis was low (r^2^ >0.98) (Fig 2A). Conversely, inter-patient variability in gene expression profiles was observed, demonstrating nanoString is suitable for discerning differences in gene expression between patients (Fig 2B). These data demonstrate the nanoString platform as a robust platform for gene expression evaluation in FFPE sqNSCLC tissues.

### Expression of multiple genes on the *FGFR1* locus is significantly higher in amplified sqNSCLC samples

**Fig 3:**
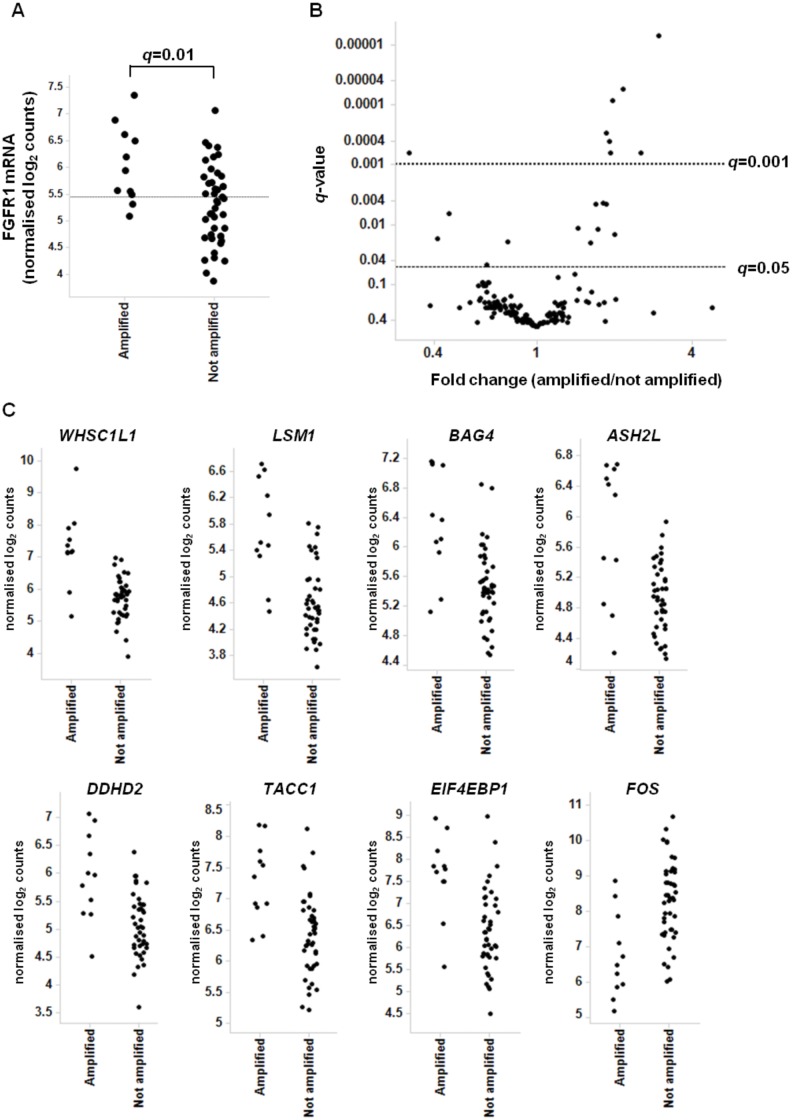
Identification of genes with significant expression changes between *FGFR1* amplified and non-amplified sqNSCLC tissues. A) FGFR1 mRNA expression was assessed by nanoString in *FGFR1* amplified and non-amplified tissues (as determined by FISH). Normalized log_2_ values are shown for each sample. Horizontal line indicates the mean *FGFR1* expression of all samples. B) Volcano plot identifying genes with statistically significant expression changes between the amplified and non-amplified cohorts. C) nanoString gene expression data is shown for each gene with *q*-value <0.0001 between amplified and non-amplified cohorts in B. Normalized log_2_ values are shown.

To characterize the relationship between *FGFR1* gene amplification and expression in sqNSCLC tissues, a panel of 90 tumour samples was profiled for *FGFR1* amplifications by FISH analysis using a specific *FGFR1* gene probe (RP11-957P17 Chr8: 38255823–38443297 (GRCh37/hg19)). A total of 13/90 sqNSCLC samples (14.4%) were confirmed as *FGFR1* gene amplified (defined as an *FGFR1/CEN-8* gene probe ratio of ≥2 or cluster signals in ≥10% of tumour cells) (S1 Table). 55 of these samples, including 11 *FGFR1* amplified samples, were analyzed for *FGFR1* mRNA expression on the nanoString platform. The nanoString codeset also contained other 8p12 amplicon genes, FGF receptors and ligands as well as additional genes of interest (S2 Table). *FGFR1* expression was significantly higher in the amplified samples than in the non-amplified samples (*q* = 0.012). Amplification enriched for the highest expressing samples, with the majority of amplified samples (82%) having *FGFR1* expression levels greater than the mean. However, there was significant overlap in *FGFR1* expression between the amplified and non-amplified cohort, with several non-amplified tumours observed to have expression levels equivalent to those of the amplified tumours (Fig 3A). Although overall a poor correlation was observed between FGFR1 RNA and protein expression (data not shown), when samples were divided into those with RNA expression levels below and above the mean protein level was significantly higher in those samples with FGFR1 expression above the mean (S4A Fig). FGFR1 protein expression by IHC revealed a similar pattern of overlapping expression levels between the amplified and non-amplified tumour samples, although while FGFR1 protein was undetectable in 68% (30/44) of the non-amplified tumours this was the case for only 9% of the amplified cancers (1/11) (S4B Fig).

To identify additional differentially expressed genes between the amplified and non-amplified groups, we performed a statistical analysis on the nanoString gene expression profiles. Using a cut-off of *q*<0.05, expression of 17 additional genes were significantly associated with *FGFR1* amplification (Fig 3B). Interestingly, 11 of these are genes are *FGFR1* neighbors on the 8p12 amplicon; in particular *WHSC1L1*, *LSM1*, *BAG4*, *ASH2L*, *DDHD2*, *TACC1* and *EIF4EBP1* were significantly overexpressed in the *FGFR1* amplified samples with *q*-values less than 0.001 (Fig 3C). 4 genes were associated with lower expression in the amplified samples, *FOS*, *ARTN*, *EGR1* and *ZEB1*. Of these, only *FOS* had a *q*-value less than 0.001 (S3 Table).

**Fig 4:**
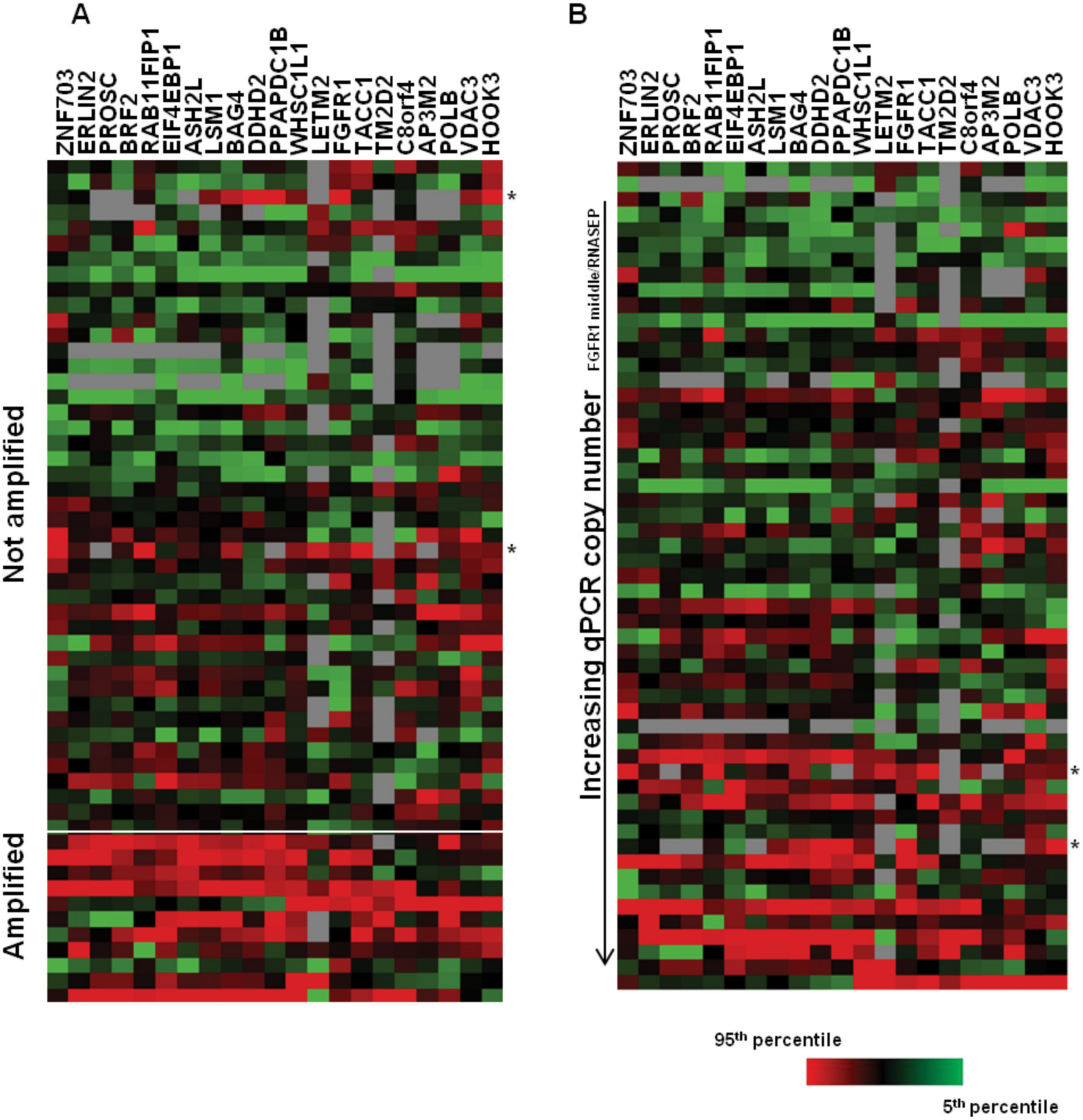
Gene expression profiles of 8p amplicon genes in sqNSCLC tissues. A) Heatmap reflecting nanoString gene expression profiles of *FGFR1* amplified and non-amplified sqNSCLC tissues as determined by FISH. * indicates non-amplified samples which had gene expression profiles similar to those of the amplified samples. B) Heatmap reflecting nanoString gene expression profiles of sqNSCLC ranked by increasing *FGFR1* inferred gene copy number as determined by qPCR. * indicates the same samples identified in A. red: 95^th^ percentile, green: 5^th^ percentile; grey: not detected.

The elevated expression of additional 8p genes in *FGFR1* amplified tumours is consistent with data suggesting that the *FGFR1* amplicon in lung cancer is a broad rather than a focal one [13]. Fig 4A shows a heatmap of 8p12 amplicon gene expression within each tumour. In accordance with the statistical analysis, *FGFR1* amplified samples showed increased expression of genes across the 8p amplicon. Interestingly, in several amplified samples the relative expression of *FGFR1* was lower than that of neighboring genes, suggesting the presence of additional regulators of *FGFR1* expression on the background of *FGFR1* amplification.

As FISH is an *in situ* technique, where 50 cells in an amplified region are counted, we sought to validate these findings using qPCR assays where DNA is isolated from the tumour samples in a manner comparable to that used for RNA extraction. Using three different qPCR assays, targeting distinct (5’, 3’ and middle) regions of the *FGFR1* gene, copy number was determined as described above. In breast tumours the 8p11-12 amplicon is broad resulting in increased copy number of *FGFR1* and of multiple neighboring genes [20] and studies have shown that some of these neighboring genes, for example *WHSC1L1*, *BAG4* and *DDHD2*, can be oncogenic [21, 22]. Although FISH and qPCR assays indicate that an amplification of the *FGFR1* gene has occurred, these do not provide information on the other affected genes. It is therefore of interest to investigate the relative expression of these genes in sqNSCLC where *FGFR1* copy number is increased. Spearman correlation analysis was used to identify genes for which expression was correlated with increasing *FGFR1* qPCR copy number. As expected, *FGFR1* expression was associated with increasing *FGFR1* inferred copy number (mean *r* = 0.305; *p* = 0.036). However, as with the FISH analysis, the *FGFR1* neighbor genes *WHSC1L1*, *DDHD2* and *BAG4* were most highly associated with increasing *FGFR1* copy number (mean *r* = 0.61, 0.58 and 0.56, respectively and *p* <0.0001) (S4 Table). This is visualized in a gene expression heatmap where samples are ordered by increasing gene copy number as determined by qPCR assay (Fig 4B). As inferred copy number increases so does expression of genes across the 8p amplicon. This implies that similar to breast cancer, the 8p11-12 amplicon in sqNSCLC extends beyond the *FGFR1* gene resulting in elevated expression of several genes, potentially contributing to oncogenesis and/or modulating the cellular response to FGFR1. Overall, good correlation between FISH and qPCR was observed, with samples amplified by FISH tending to have higher qPCR values (S5 Fig). Interestingly, in the FISH analysis the 8p gene expression heatmap revealed two non-amplified samples that had gene expression profiles similar to those of the amplified samples, including relatively high *FGFR1* expression. On analysis of the qPCR data, both of these samples had increased *FGFR1* inferred gene copy number. The discrepancy between classification of these samples as amplified or non-amplified may have been the result of inter-tumoural heterogeneity in amplification patterns.

### In-depth high resolution array CGH of clinical samples and cell lines confirms the presence of a broad amplicon at the *FGFR1* locus

The gene expression data presented here suggests the presence of a broad amplicon in the region of *FGFR1* on chromosome 8. We further explored this amplicon through in-depth high resolution genomic analysis of the *FGFR1* locus using a custom CGH array in a subset of five sqNSCLC tissues, four of which carried *FGFR1* amplification by FISH (S2 Table). Using this method, two samples had a low level *FGFR1* gain (inferred copy numbers of 2.5 and 2.9), two samples showed a higher level of amplification (inferred copy numbers of 4.9 and 4.2), and the non-amplified sample by FISH did not harbour a significant copy number alteration in *FGFR1*.

**Fig 5:**
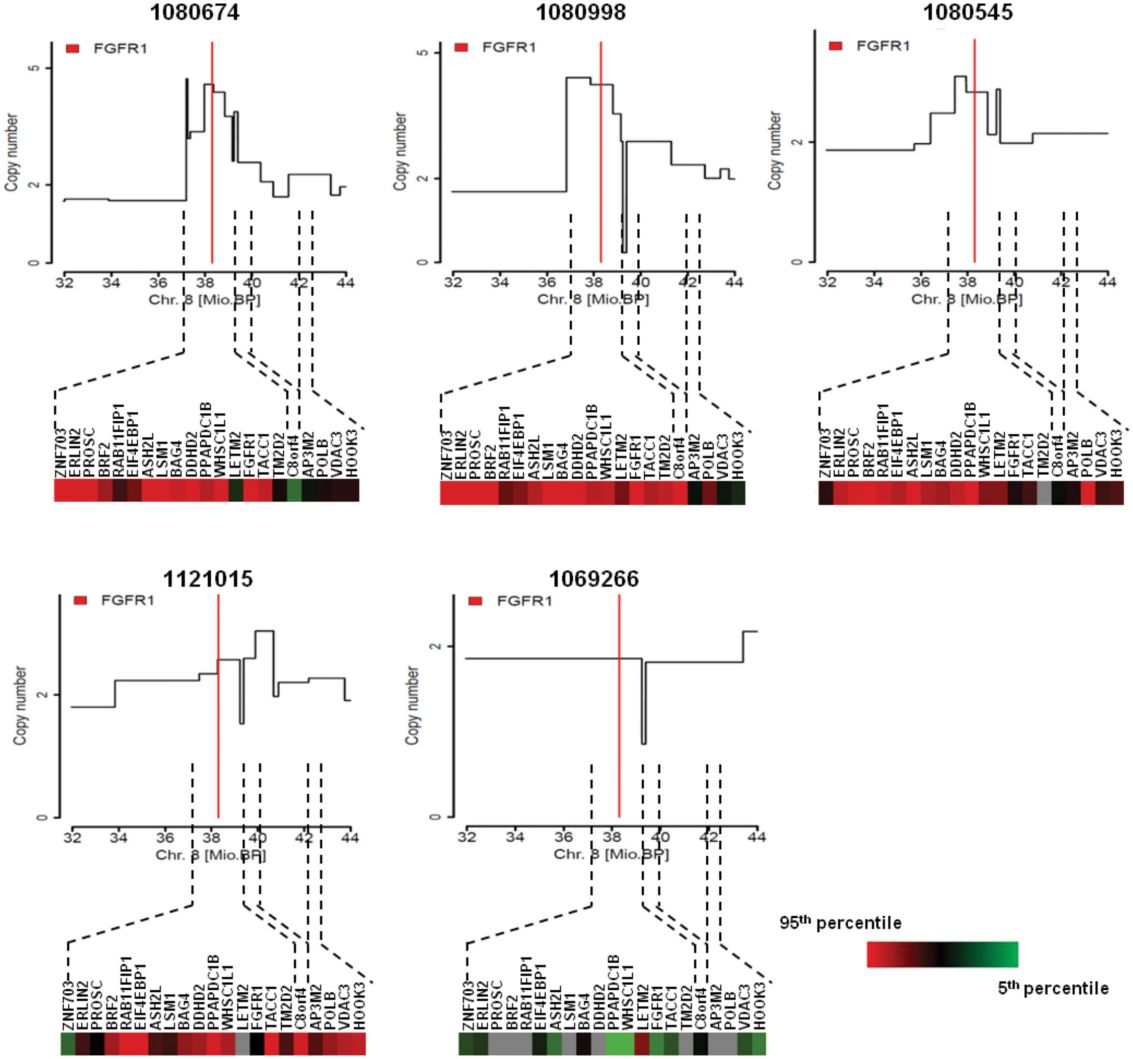
In-depth high resolution array CGH of clinical samples confirms the presence of a broad amplicon at the *FGFR1* locus. In-depth high resolution genomic analysis of the *FGFR1* locus using a custom CGH array was performed on indicated samples. The position of *FGFR1* is indicated by a red line. Corresponding nanoString gene expression profiles for genes in the chromosomal region are shown. red: 95^th^ percentile, green: 5^th^ percentile; grey: not detected.

The detailed view of the *FGFR1* locus afforded by these data demonstrated a broad and varied amplicon within clinical sqNSCLC tissues. For example, although samples 1080998 and 1080545 showed *FGFR1* copy number gains, slightly higher copy numbers were observed 300KB telomeric of *FGFR1*, where the highest peak contained the genes *ZNF703*, *ERLIN2*, *PROSC*, *GPR124*, *BRF2*, *RAB11F1P1*, *GOT1L1*, *ADRB3* and *EIF4EBP1*. This is consistent with the nanoString gene expression data for these samples, where genes towards the telomeric end of *FGFR1* showed relatively higher expression than those at the centromeric end of the amplicon. Conversely, sample 1121015 showed low level copy number gain centromeric to *FGFR1* and these genes were expressed at relatively higher levels than those telomeric to *FGFR1*. The highest peak for sample 1080674 contained full-length *FGFR1* (copy number 4.58) and included other genes such as *ASH2L*, *LSM1*, *WHSC1L1*, *LETM2*, *C8orf86*, *RNF5P1*, *TACC1*, and *PLEKHA2*, with an inferred copy number of 4.3 across this whole region (Fig 5).

**Fig 6:**
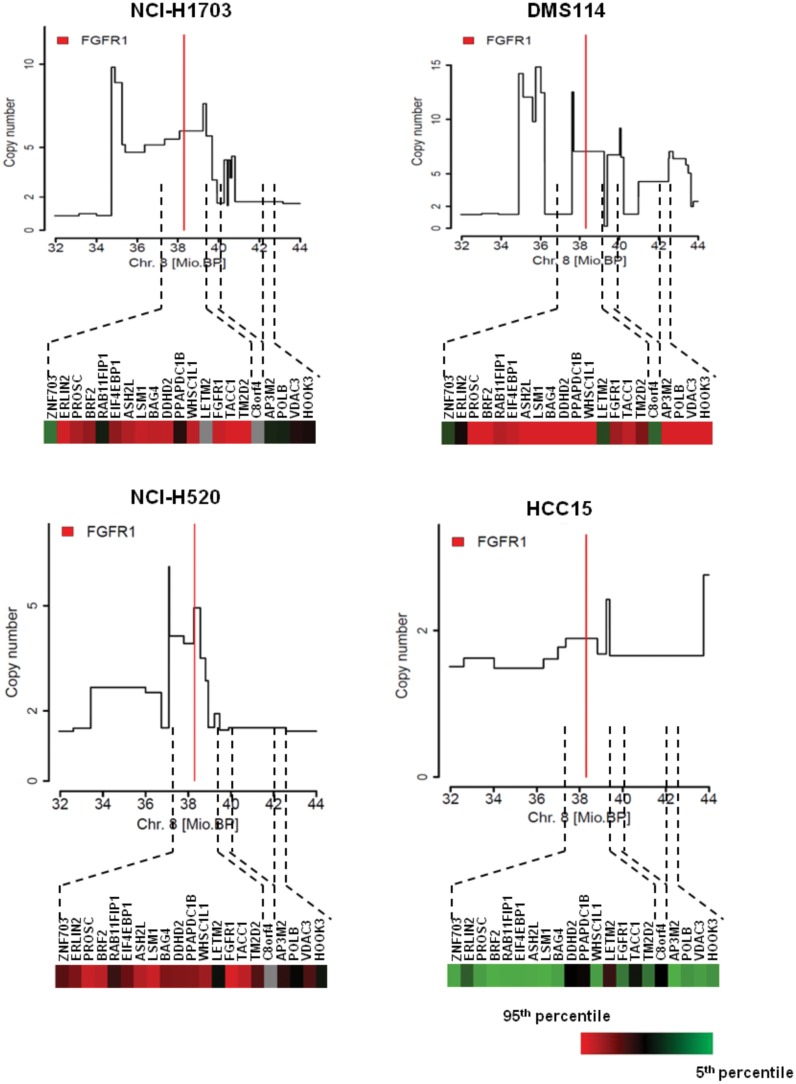
In-depth high resolution array CGH of cell lines identifies a broad amplicon at the *FGFR1* locus. In-depth high resolution genomic analysis of the *FGFR1* locus using a custom CGH array was performed on indicated cell lines. The position of *FGFR1* is indicated by a red line. Corresponding nanoString gene expression profiles for genes in the chromosomal region are shown. red: 95^th^ percentile, green: 5^th^ percentile; grey: not detected.

We also carried out in-depth analysis of the *FGFR1* locus in a subset of lung cancer cell lines to determine whether the amplicon structure differs between pre-preclinical and clinical models. NCI-H1703 and NCI-H520 cells showed an *FGFR1* gain (inferred copy numbers of 5.9 and 5.0, respectively), which was found in the same amplicon as the genes *LSM1*, *BAG4*, *DDHD2*, *PPAPDC1B*, *WHSC1L1*, *LETM2*, *C8orf86*, *RNF5P1*, *TACC1*, *PLEKHA2*, *HTRA4*, *TM2D2*, *ADAM9* and *ADAM32*, telomeric to *FGFR1*. Consistent with these amplicon profiles, genes were not overexpressed at the centromeric end of the *FGFR1* locus in NCI-H1703 or NCI-H520. These cell lines therefore gave profiles similar to that seen for the clinical samples 1080998 and 1080674 above. DMS114 showed a heterogeneous architecture across the whole *FGFR1* locus. The broad amplicon was defined by a focal peak with an inferred copy number of 12.5 (containing the genes *ERLIN2* and *PROSC*), followed by a plateau containing the genes *WHSC1L1*, *LETM2* and *FGFR1* at an inferred copy number of 7. Additional focal and broad peaks were detected at the centromeric end of the *FGFR1* locus. Consistent with a very broad amplicon being present in these cells, gene expression analysis revealed relatively high expression of all 8p amplicon genes tested in this cell line. The non-amplified cell line HCC15 showed two minor gains ranging from an inferred copy number 2.4 and 2.7, both not containing *FGFR1* (Fig 6). This analysis therefore revealed the *FGFR1* amplicon has a similar broad and heterogeneous structure in cell-lines as in clinical samples.

## Discussion

In preclinical models of sqNSCLC, several studies revealed *FGFR1* as a driving oncogene, and small molecule mediated inhibition of FGFR signalling inhibited growth of *in vivo* models [10]. However, clinical responses with FGFR inhibitors in patients with *FGFR1* amplifications have been broadly disappointing. It is key to try to understand this disconnect from preclinical to clinical studies and apply learning to future programs. To this end, we undertook a comparison of the expression profiles of preclinical models to those of clinical sqNSCLC samples. Cell lines with an *FGFR1* amplification overexpressed *FGFR1* at both mRNA and protein levels and this overexpression resulted in sensitivity to the FGFR inhibitor AZD4547. Further, both LK-2 and NCI-H226 cells which did not harbour an *FGFR1* amplification also overexpressed FGFR1 and were sensitive to FGFR inhibition. Gene expression analysis of clinical tissue similarly demonstrated that *FGFR1* amplification resulted in significantly higher expression of *FGFR1* mRNA. However, there was significant overlap in *FGFR1* expression levels between amplified and non-amplified samples. This is consistent with the observation that increased mRNA expression by mRNA ISH occurs in the absence of *FGFR1* amplification in primary lung tumours and *FGFR1* mRNA is not elevated in all amplified samples [16]. Other studies have reported that only a subset of *FGFR1* amplified sqNSCLC tissues express high levels of FGFR1 mRNA [23, 24], or FGFR1 protein [24, 25]. In addition in a panel of head and neck squamous cell carcinoma cell lines, FGFR1 mRNA and protein were better predictors of response to the FGFR inhibitor BGJ398 than *FGFR1* copy number [26]. We have previously shown that a patient-derived tumour xenograft with *FGFR1* amplification but low level FGFR1 protein expression was less sensitive to AZD4547 treatment than models with co-occuring FGFR1 amplification and high FGFR1 protein expression [10]. Taking these data together, it is tempting to speculate that selection of patients based on *FGFR1* expression rather than amplification could provide a better approach for trials of FGFR inhibitors. However, it is challenging to define meaningful cut-off criteria for high expression. If high *FGFR1* RNA expression was to be defined as levels equivalent to those observed in AZD4547 sensitive preclinical models, then only 2 of 11 amplified patients from this sample set would be eligible for entry onto the trial, as expression levels seen in the clinical tissue did not reach those seen in the overexpressing cell lines for the majority of samples. Interestingly, this was not the case for *WHSC1L1*, where many amplified tumours expressed *WHSC1L1* to levels equivalent to those in amplified and sensitive cell lines (S6 Fig). Similarly analysis of FGFR1 protein expression by IHC revealed a range of overlapping expression levels in amplified and non-amplified tumours. This suggests that in tumour samples additional transcriptional and translational mechanisms may alter *FGFR1* expression in amplified samples.

It is clear that the *FGFR1* amplicon has a broad and heterogeneous structure in clinical lung tumours resulting in high expression of multiple genes contained within the amplicon and this may well be expected to influence response to FGFR inhibition. However the FGFR inhibitor sensitive cell lines have a similar broad and hetergenous amplicon expression indicating that expression of co-amplied genes does not necessarily lead to resistance to FGFR inhibition.

Although trials with FGFR inhibitors have been widely disappointing, there have been reports of patients receiving benefit from these drugs. Indeed, the biology is complex and may mask the true biomarker of response. It is clearly necessary to carry out a range of biomarker analyses to develop our understanding of determinants of response and resistance to FGFR inhibition, including direct sequencing of *FGFR1* as well as potential modulators of FGFR signaling. In this regard, the multiplexed nature of the nanoString platform for assessing gene expression in clinical lung samples provides a surrogate for amplicon breadth by determining relative expression of genes across the amplicon. Furthermore, patient selection for trials of FGFR inhibitors has been carried out on tumour tissue taken at the time of diagnosis. Analysis of tumour tissue at the time of treatment may reveal temporal heterogeneity in *FGFR1* amplification and expression which alters the predicted response to FGFR inhibitors.

## Supporting Information

S1 Fig Response of sqNSCLC cell lines to AZD4547 in a clonogenic assay. Indicated cell lines were treated with DMSO control or a dose response of AD4547 for 21 days. Cells were fixed and stained with crystal violet to identify colonies.
(PPTX)

S2 Fig Expression of FGFR2 and FGFR3 mRNA in sqNSCLC cell line panel by nanoString analysis. (PPTX)

S3 Fig Comparison of mRNA expression by Taqman RT-PCR and nanoString. mRNA isolated from 1 x 5um FFPE slide of sqNSCLC tissue from 12 patients was profiled for indicated genes on nanoString and by Taqman RT-PCR. Data was normalised to housekeeping controls and is plotted as normalised log_2_ counts (Nanostring) or -dCT (Taqman). Samples detected above the limit of detection on both platforms are shown. Bars: stdev Taqman replicates.
(PPTX)

S4 Fig Analysis of FGFR1 protein expression in sqNSCLC cohort by immunohistochemistry. A. Samples were divided into those with FGFR1 mRNA above or below the mean by nanoString. FGFR1 protein expression (H-score) was significantly higher in samples with FGFR1 mRNA levels above the mean. B. FGFR1 protein expression was significantly higher in *FGFR1* amplified samples than non-amplified samples (as determined by FISH), with FGFR1 protein undetectable in 68% (30/44) of the non-amplified samples and 9% (1/11) of the amplified samples.
(PPTX)

S5 Fig Inferred *FGFR1* qPCR copy number in amplified and not amplified samples as determined by FISH. Inferred *FGFR1* copy number was calculated in sqNSCLC using a qPCR assay targeting the middle region of the *FGFR1* gene and RNASE P control gene. Graph indicates inferred copy number in amplified and not amplified cohorts as determined by FISH.
(PPTX)

S6 Fig *FGFR1* and *WHSC1L1* expression in sqNSCLC clinical tissue and cell lines. Red line indicates the expression cut-off for AZD4547 sensitive lines (for *FGFR1)* or for *FGFR1* amplified lines (for *WHSC1L1)*.
(PPTX)

S1 Table *FGFR1* FISH scoring for sqNSCLC cohort. (XLSX)

S2 Table nanoString codeset genes and probe sequences. (XLSX)

S3 Table Statistical analysis of expression changes between amplified and non-amplified cohorts. *q*-values were used to identify statistically significant changes between the amplified and non-amplified cohorts.
(XLSX)

S4 Table Spearman correlation analysis of association between copy number and gene expression. (XLSX)
