# Supplementary figures and images for: Characterization of FGFR1 Locus in sqNSCLC Reveals a Broad and Heterogeneous Amplicon
Source: PLoS One. 2016 Feb 23;11(2):e0149628. doi: 10.1371/journal.pone.0149628 (PMC4764357; doi:10.1371/journal.pone.0149628)

## Slide 1
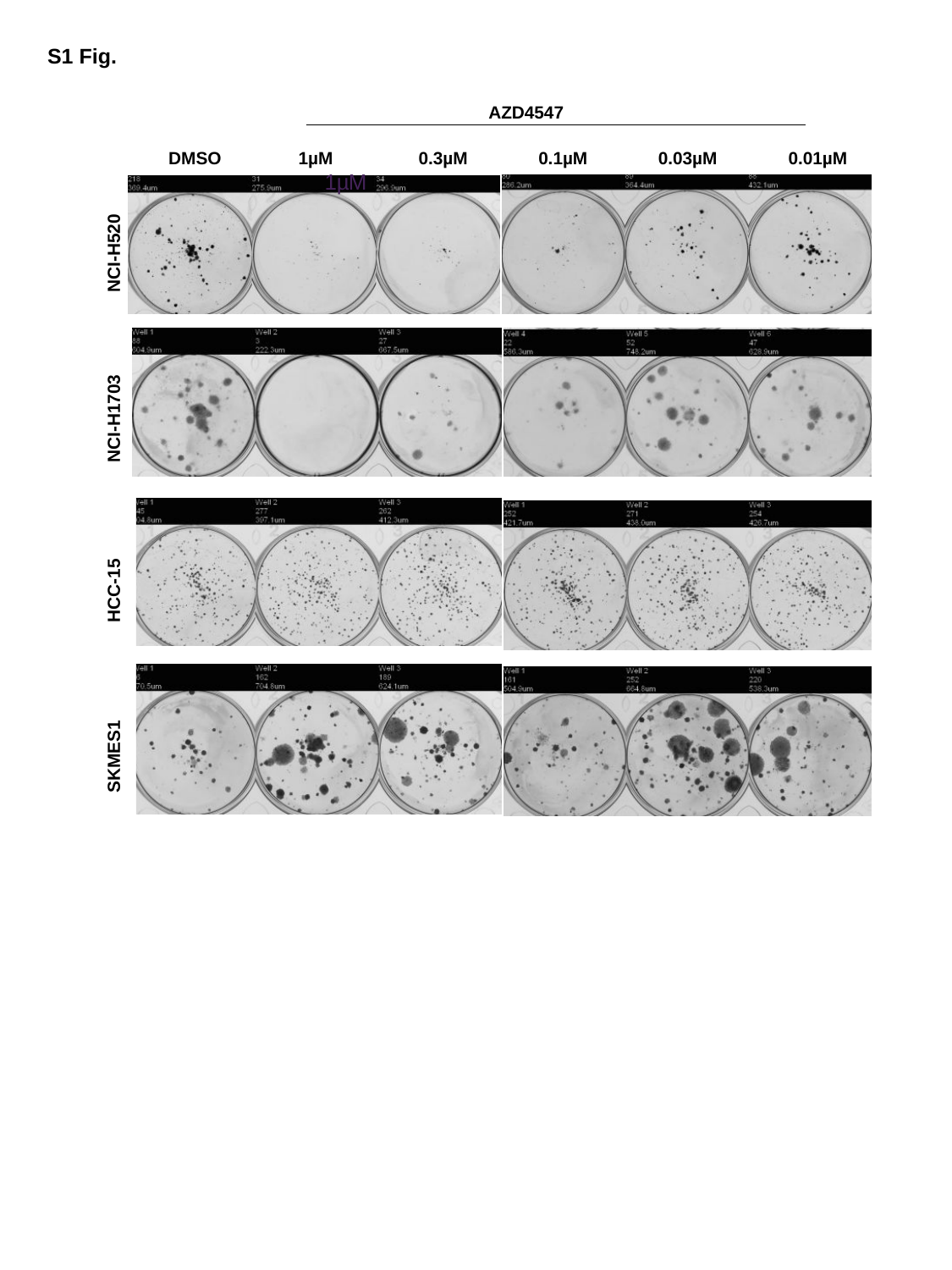

S1 Fig.
AZD4547
DMSO
1µM
0.3µM
0.1µM
0.03µM
0.01µM
NCI-H520
NCI-H1703
HCC-15
SKMES1

Supplement: S1 Fig — Indicated cell lines were treated with DMSO control or a dose response of AD4547 for 21 days. Cells were fixed and stained with crystal violet to identify colonies. (PPTX) [file pone.0149628.s001.pptx]

## Slide 1
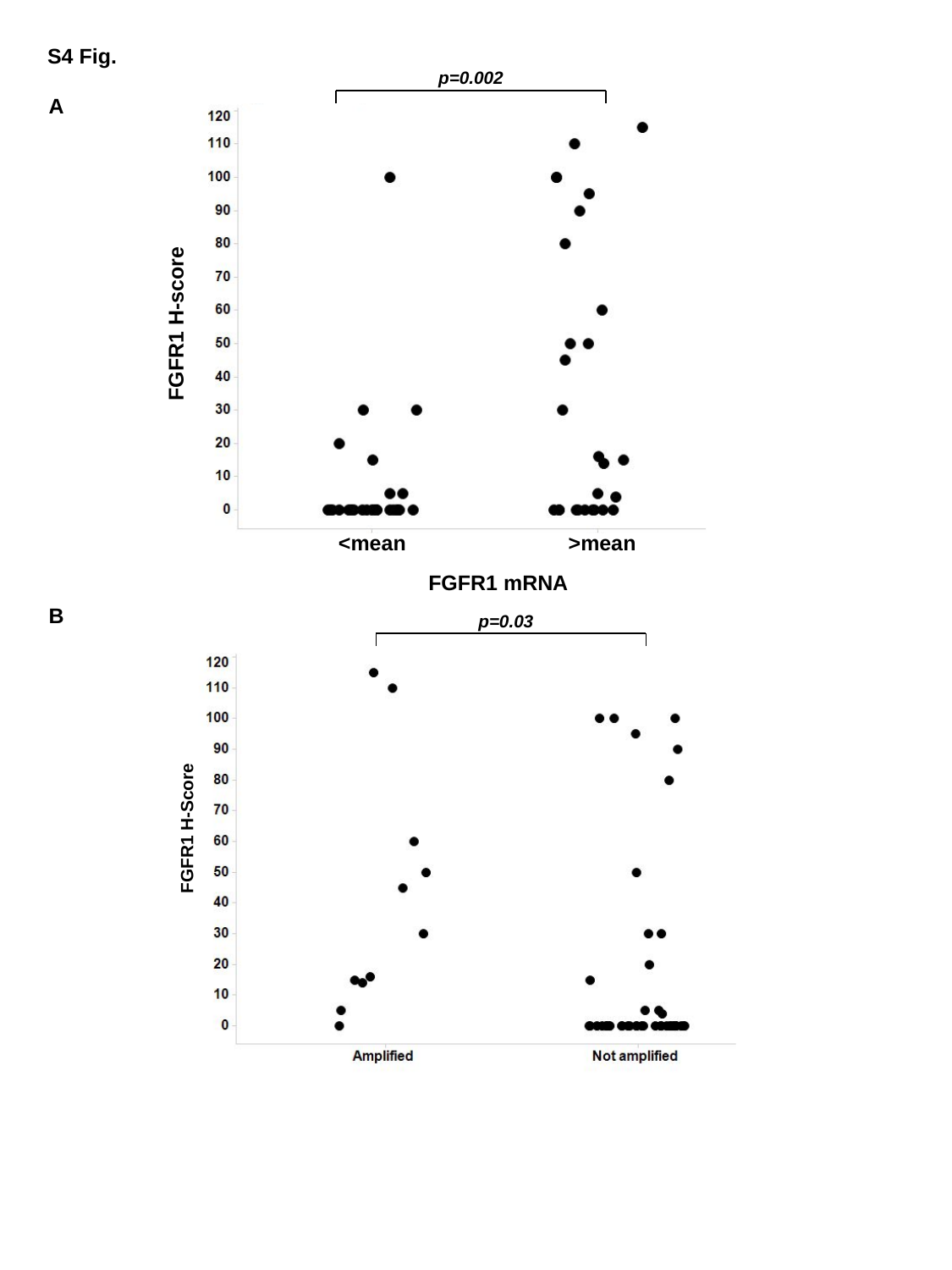

S4 Fig.
p=0.002
A
FGFR1 H-score
<mean
>mean
FGFR1 mRNA
B
p=0.03
FGFR1 H-Score

Supplement: S4 Fig — A. Samples were divided into those with FGFR1 mRNA above or below the mean by nanoString. FGFR1 protein expression (H-score) was significantly higher in samples with FGFR1 mRNA levels above the mean. B. FGFR1 protein expression was significantly higher in FGFR1 amplified samples than non-amplified samples (as determined by FISH), with FGFR1 protein undetectable in 68% (30/44) of the non-amplified samples and 9% (1/11) of the amplified samples. (PPTX) [file pone.0149628.s004.pptx]

## Slide 1
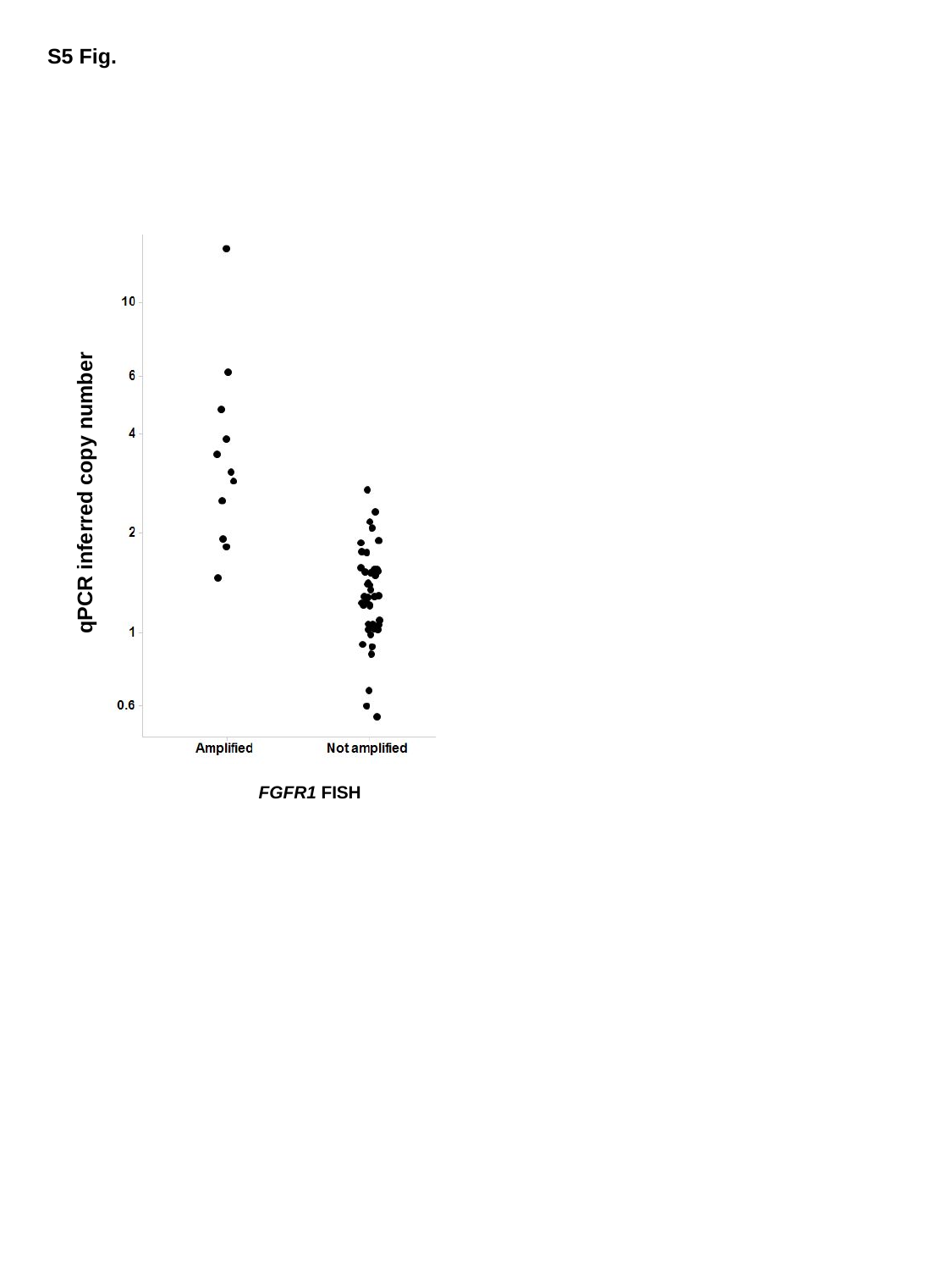

S5 Fig.
qPCR inferred copy number
FGFR1 FISH

Supplement: S5 Fig — Inferred FGFR1 copy number was calculated in sqNSCLC using a qPCR assay targeting the middle region of the FGFR1 gene and RNASE P control gene. Graph indicates inferred copy number in amplified and not amplified cohorts as determined by FISH. (PPTX) [file pone.0149628.s005.pptx]
