# Supplementary material for: Characterization of FGFR1 Locus in sqNSCLC Reveals a Broad and Heterogeneous Amplicon
Source: PLoS One. 2016 Feb 23;11(2):e0149628. doi: 10.1371/journal.pone.0149628 (PMC4764357; doi:10.1371/journal.pone.0149628)
Supplement: S2 Fig — (PPTX) [file pone.0149628.s002.pptx]

## Slide 1
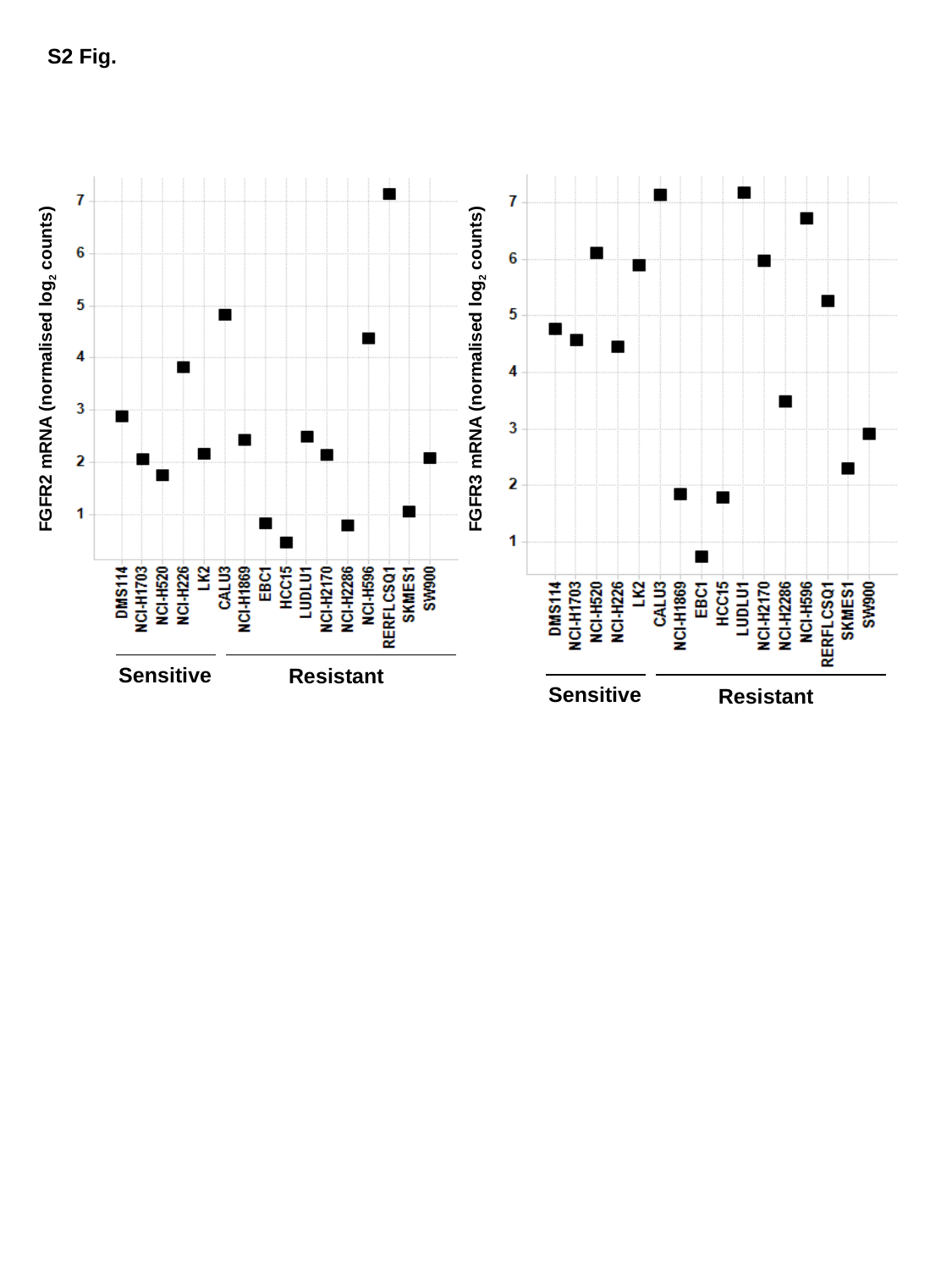

S2 Fig.
FGFR2 mRNA (normalised log2 counts)
FGFR3 mRNA (normalised log2 counts)
Sensitive
Resistant
Sensitive
Resistant
