# Supplementary material for: Characterization of FGFR1 Locus in sqNSCLC Reveals a Broad and Heterogeneous Amplicon
Source: PLoS One. 2016 Feb 23;11(2):e0149628. doi: 10.1371/journal.pone.0149628 (PMC4764357; doi:10.1371/journal.pone.0149628)
Supplement: S3 Fig — mRNA isolated from 1 x 5um FFPE slide of sqNSCLC tissue from 12 patients was profiled for indicated genes on nanoString and by Taqman RT-PCR. Data was normalised to housekeeping controls and is plotted as normalised log2 counts (Nanostring) or -dCT (Taqman). Samples detected above the limit of detection on both platforms are shown. Bars: stdev Taqman replicates. (PPTX) [file pone.0149628.s003.pptx]

## Slide 1
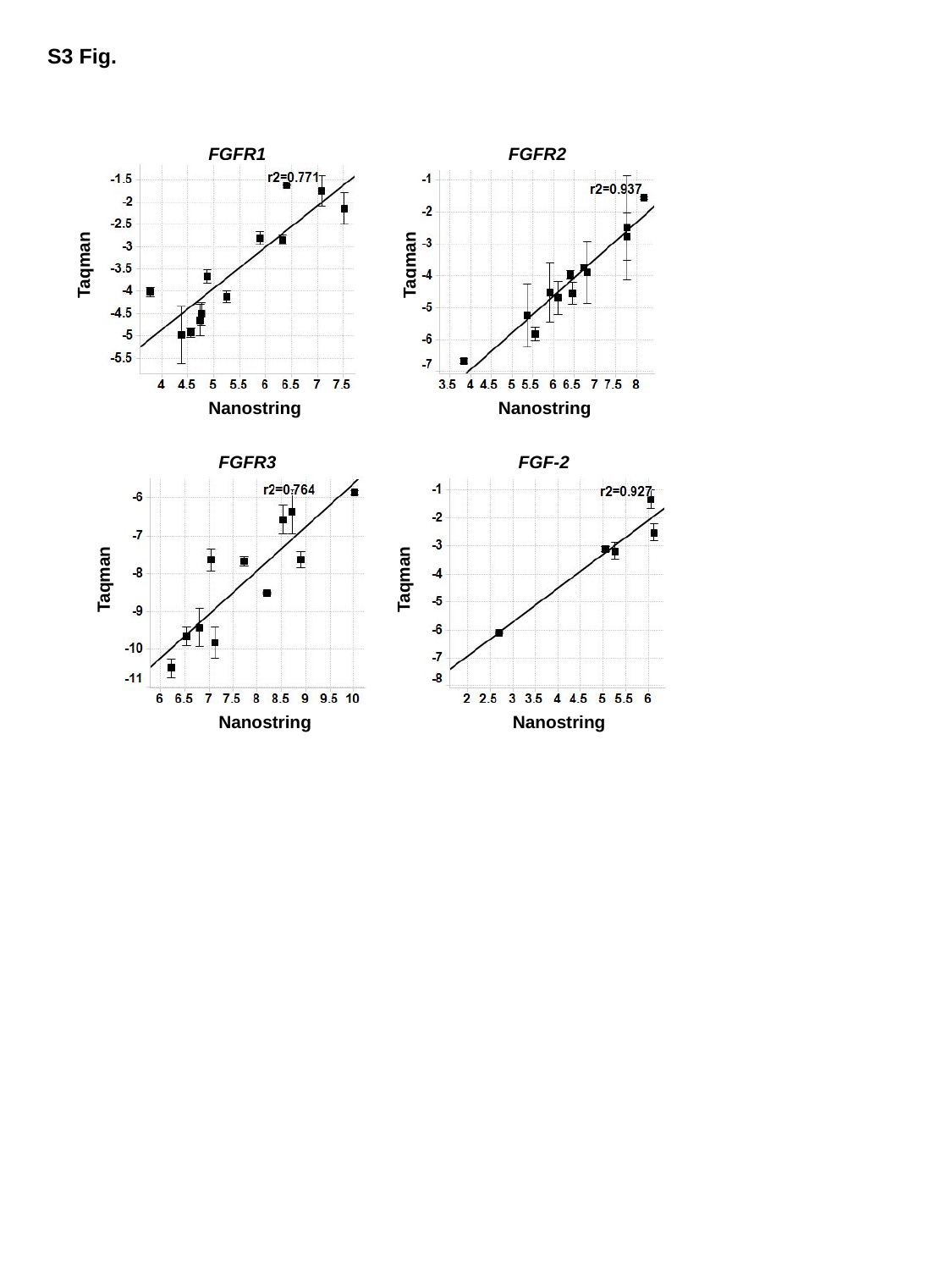

S3 Fig.
FGFR1
FGFR2
Taqman
Taqman
Nanostring
Nanostring
FGFR3
FGF-2
Taqman
Taqman
Nanostring
Nanostring
