# Supplementary material for: Characterization of FGFR1 Locus in sqNSCLC Reveals a Broad and Heterogeneous Amplicon
Source: PLoS One. 2016 Feb 23;11(2):e0149628. doi: 10.1371/journal.pone.0149628 (PMC4764357; doi:10.1371/journal.pone.0149628)
Supplement: S6 Fig — Red line indicates the expression cut-off for AZD4547 sensitive lines (for FGFR1) or for FGFR1 amplified lines (for WHSC1L1). (PPTX) [file pone.0149628.s006.pptx]

## Slide 1
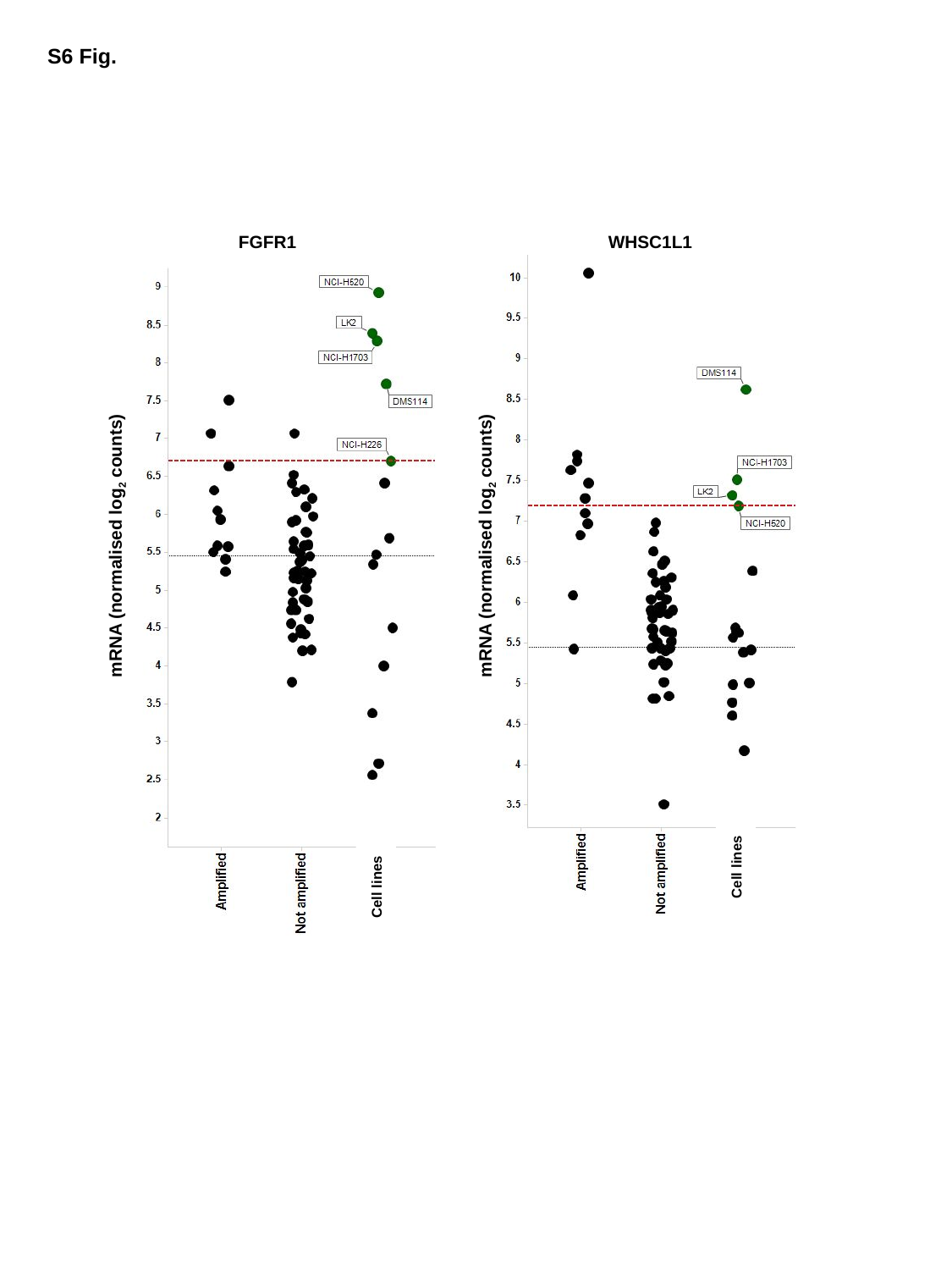

S6 Fig.
FGFR1
WHSC1L1
 mRNA (normalised log2 counts)
 mRNA (normalised log2 counts)
 Cell lines
 Cell lines
